# Amplitude-based gated phase-controlled rescanning in carbon-ion scanning beam treatment planning under irregular breathing conditions using lung and liver 4DCTs

**DOI:** 10.1093/jrr/rru032

**Published:** 2014-05-15

**Authors:** Shinichiro Mori, Taku Inaniwa, Takuji Furukawa, Wataru Takahashi, Mio Nakajima, Toshiyuki Shirai, Koji Noda, Shigeo Yasuda, Naoyoshi Yamamoto

**Affiliations:** Research Center for Charged Particle Therapy, National Institute of Radiological Sciences, Inage-ku, Chiba 263-8555, Japan

**Keywords:** carbon ion beam, 4DCT, irregular breathing, lung, liver, scanning beam, treatment planning

## Abstract

Amplitude-based gating aids treatment planning in scanned particle therapy because it gives better control of uncertainty with the gate window. We have installed an X-ray fluoroscopic imaging system in our treatment room for clinical use with an amplitude-based gating strategy. We evaluated the effects of this gating under realistic organ motion conditions using 4DCT data of lung and liver tumors. 4DCT imaging was done for 24 lung and liver patients using the area-detector CT. We calculated the field-specific target volume (FTV) for the gating window, which was defined for a single respiratory cycle. Prescribed doses of 48 Gy relative biological effectiveness (RBE)/fraction/four fields and 45 Gy RBE/two fractions/two fields were delivered to the FTVs for lung and liver treatments, respectively. Dose distributions were calculated for the repeated first respiratory cycle (= planning dose) and the whole respiratory data (= treatment dose). We applied eight phase-controlled rescannings with the amplitude-based gating. For the lung cases, D95 of the treatment dose (= 96.0 ± 1.0%) was almost the same as that of the planning dose (= 96.6 ± 0.9%). D_max_/D_min_ of the treatment dose (= 104.5 ± 2.2%/89.4 ± 2.6%) was slightly increased over that of the planning dose (= 102.1 ± 1.0%/89.8 ± 2.5%) due to hot spots. For the liver cases, D_95_ of the treatment dose (= 97.6 ± 0.5%) was decreased by ∼ 1% when compared with the planning dose (= 98.5 ± 0.4%). D_max_/D_min_ of the treatment dose was degraded by 3.0%/0.4% compared with the planning dose. Average treatment times were extended by 46.5 s and 65.9 s from those of the planning dose for lung and liver cases, respectively. As with regular respiratory patterns, amplitude-based gated multiple phase-controlled rescanning preserves target coverage to a moving target under irregular respiratory patterns.

## INTRODUCTION

Of more than 7000 patients treated with passive scattering carbon-ion beam in our hospital since 1994, ∼ 40% of cases included a respiratory gating strategy [1]. In 2011, we started carbon-ion scanning beam therapy for a limited range of anatomical sites not requiring respiratory gating [2], and we are now preparing to extend respiratory gating treatment to the thoracic and abdominal regions. Given that the patient respiratory pattern is not always reproducible [3], dose conformation to the target under irregular breathing conditions is an important issue. 4DCT plays an important role in radiotherapy treatment planning for moving organs, but while current commercially available treatment-planning systems can import 4DCT data, they cannot as yet evaluate 4D dose distribution, including deformable image registration (DIR). Calculation of dose distribution requires 4DCT image data over all respiratory cycles. This is not suitable for radiological protection because, even though patients receive particle beam treatment, longer 4DCT imaging over a single respiratory cycle increases radiation dose. Nevertheless, 4D dose distribution assessment under an irregular breathing pattern is required before scanning irradiation to moving organs can be commenced.

Our scanning beam treatment for moving organs involves phase-controlled rescanning. We are also ready to implement tumor amplitude-based gating, which allows targeting of the treatment beam to the tumor position as defined in treatment planning.

Here, to extend understanding of organ motion during regular respiration to motion during irregular respiration under more clinical conditions, we have evaluated carbon-ion scanning dose distributions with irregular respiration using lung/liver 4DCT datasets.

## MATERIALS AND METHODS

### Patients and imaging

A total of 24 subjects (mean age: 70.8 years, standard deviation: 11.1 years) randomly selected from inpatients undergoing actual conventional passive carbon-ion beam treatment in our hospital (Table 1) provided informed consent to participate. Of these patients, 14 had lung cancer and 10 had hepatocellular carcinoma (HCC). Relevant patient demographics, tumor pathology, size, tumor location, gross tumor volume (GTV), center of mass (COM) etc. are listed in Table 1. 4DCT was performed with the area-detector CT (ADCT) (= 256 multi-slice CT) under free-breathing conditions, with patient respiration monitored using an external respiratory-sensing system, which consists of a position-sensitive detector and an infrared-emitting light termed an ‘active’ marker [4]. For liver 4DCT, we acquired a second 4DCT scan after completion of the first by moving the couch to the next position, with an overlap region of ∼ 2 cm, on the basis that this scan range is insufficient for whole liver imaging ( ∼ 13 cm scan region in a single rotation). A resorting process was adopted for the first and second 4DCT scans, although resorting as a function of respiratory phase was simpler than for a conventional multislice CT because there were only two resorting datasets. Pixel size and slice thickness were 0.78 mm and 1.0 mm, respectively. Generally, the 4DCT datasets were subdivided into 10 phases (T00, peak inhalation; T50, peak exhalation) based on the phase of the respiratory signal. We further subdivided these into 20 phases based on the amplitude signal.

**Table 1.** Patient characteristics

									Respiratory cycle		Respiratory peak position ratio	
Pt. no.	Gender	Age (year)	Location		Pathology	Position	DIR accuracy (mm)	3D-COM (mm)	Planning	Treatment	Inhalation	Exhalation
1	M	75	Lung	S4	ADC	SP	1.8	2.9	4.1	4.2	0.9	−0.1
2	M	84	Lung	S3	SCC	SP	0.9	1.7	3.9	3.5	0.5	−0.3
3	M	76	Lung	S6	meta	PR	1.7	5.5	3.8	3.7	0.9	−0.3
4	F	77	Lung	S3	SCC	SP	1.8	4.0	3.3	2.8	1.0	0.0
5	F	48	Lung	S10	meta	PR	1.7	12.1	2.9	2.7	0.8	0.5
6	F	58	Lung	S7	meta	SP	2.2	6.4	4.0	4.1	1.0	−0.1
7	M	65	Lung	S9	SCC	PR	2.0	13.4	4.3	3.9	0.9	1.2
8	F	80	Lung	S3	ADC	SP	1.0	2.7	4.2	4.0	1.0	0.0
9	M	75	Lung	S10	SCC	PR	2.0	12.4	3.1	2.9	0.9	−0.1
10	F	81	Lung	S3	meta	SP	1.3	3.4	2.7	2.5	0.8	0.0
11	M	64	Lung	S4	SCC	SP	1.3	4.9	4.4	2.6	1.1	0.1
12	M	61	Lung	S8	ADC	PR	1.5	7.7	3.6	3.2	1.1	0.1
13	M	80	Lung	S5	ADC	SP	1.1	2.1	3.4	3.3	1.0	0.0
14	F	79	Lung	S9	ADC	PR	1.3	5.0	2.8	2.9	1.0	−0.2
15	M	82	Liver	S7	HCC	PR	1.8	6.7	3.0	2.5	0.9	0.0
16	M	54	Liver	S8	HCC	SP	1.5	17.2	5.8	8.2	1.1	−0.1
17	F	74	Liver	S7	HCC	PR	1.3	12.4	2.8	2.5	1.0	−0.2
18	M	87	Liver	S8	HCC	PR	1.5	14.4	3.5	2.7	0.9	0.0
19	M	55	Liver	S6–7	HCC	PR	1.6	18.7	3.6	3.1	1.2	0.0
20	F	68	Liver	S8	HCC	PR	1.5	11.2	4.0	3.4	1.1	−0.1
21	F	65	Liver	S7	meta	SP	1.3	14.4	3.7	2.4	1.1	0.0
22	M	79	Liver	S7	meta	PR	1.2	11.4	3.7	3.6	1.2	0.0
23	M	78	Liver	S6–7	HCC	PR	1.0	12.4	4.5	2.6	1.0	0.1
24	M	54	Liver	S4	HCC	SP	1.6	15.1	4.4	2.4	0.9	−0.1
Mean		70.8 ± 11.1					1.5 ± 0.3	9.1 ± 5.2	3.7	3.3	1.0	0.0

Pt. no. = patient number, DIR = deformable image registration, 3D–COM = 3D center-of-mass, SP = supine, PR = prone, ADC = adenocarcinoma, SCC = squamous cell carcinoma, S.D. = standard deviation.

### Treatment beam irradiation techniques

The hybrid depth carbon-ion scanning method uses a mini ridge filter to create a 3-mm spread-out Bragg peak (SOBP), range shifter and 11 synchrotron energies (140–430 MeV/u) [5]. We used the same spot size (lateral scatter (P80–20) of 5.0 mm) and spacing of 2 mm in all iso-energy layers. Scanning spot speed was 100 mm/ms and 50 mm/ms for the superior–inferior and left–right directions, respectively. Maximum beam intensity was set to 1.5 × 10^8^ particles per second (pps). Range shifter and synchrotron energy were changed in 3-mm and 30-mm range steps, and change times were set to 420 ms and 150 ms, respectively [6]. We applied the layered phase-controlled rescanning (PCR) method, which performs the rescanning of all beam spots of a layer within a single gating window. After finishing one layer, the energy/range is changed and the next layer is irradiated. This process is repeated until all spots of the entire set of layers are delivered. To achieve this strategy, the dose rate for respective iso-energy layers is calculated from the gating window time and is set by the irradiation system before the delivery of each layer. Therefore, once the treatment has been started, the irradiation pattern (beam spot, dose rate etc.) cannot be changed, even though the patient respiratory pattern may have changed.

### Treatment planning

#### Countering

Radiation oncologists input the target and normal tissue contours on the reference phase (mid-exhalation (= T30) for liver and peak exhalation (= T50) for lung cases). Since respiration-induced liver displacement is large, the choice of mid-exhalation phase was approximated as the average tumor position during a respiratory cycle, which is useful in minimizing DIR errors. These contours were transferred to other phases by applying B-spline-based DIR, with a registration accuracy of 1.5 mm (1.5 mm for lung cases and 1.4 mm for liver cases). This registration accuracy was evaluated as follows: 30 landmarks points were marked on the CT data at the reference phase at peak inhalation and peak exhalation in the same coordinate system. Warping of the landmarks was performed, and the statistics for vector differences were calculated. Clinical target volume (CTV) was defined by adding a 10-mm margin to the GTV. We calculated field-specific target volume (FTV), which is considered to represent intrafractional beam range variation, using 4DCT datasets for a full single respiratory cycle (T00–T95) to emphasize tumor motion [7]. To calculate FTV, beam ranges were calculated from the beam entrance of the patient surface to the distal and proximal edges of the target for the respective phases. The FTV was then calculated by selecting the minimum and maximum beam ranges, ray by ray.

#### Planning dose distribution

**Fig. 1.**
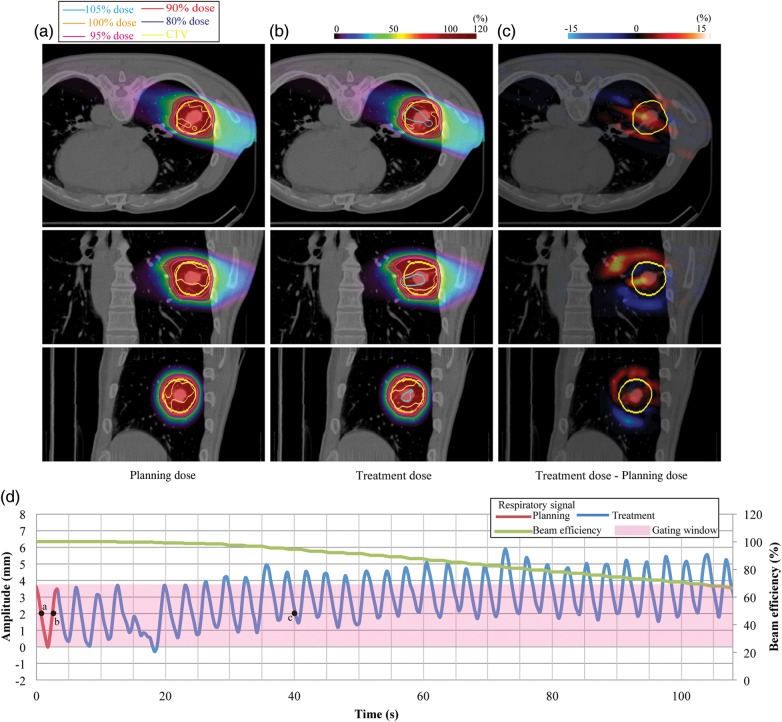
Lung dose distributions from 110° for (**a**) planning dose, (**b**) treatment dose, and (**c)** dose differences (Patient no. 13). (**d**) Respiratory wave data. Planning respiratory cycle: 3.6 s, treatment respiratory cycle: 3.1 ± 0.6 s.

Our previous study using a numeric lung phantom revealed that PCR more than four times improves dose conformation to the target [8], and we accordingly did PCR eight times in this study. No set-up uncertainty was considered. Beam weight maps for the respective FTVs were optimized by using the relative biological effectiveness (RBE)-weighted absorbed dose defined in ICRU Report 78 [9] with the first respiratory cycle data obtained during treatment. We assumed in this study that respiratory data correlated well with tumor position. When the period of respiratory data was shorter than that of scanning irradiation, we repeated the acquisition of respiratory data until scanning irradiation was completed. Since, from our clinical experiences using passive scattering irradiation [10, 11], hypofractionation treatment of the lung and liver gave a clinically acceptable level of dose to the normal tissues, total prescribed doses of 48 Gy RBE/fraction (= 12 Gy RBE/fraction via four different beam ports from the ipsilateral rather than contralateral side of the tumor) and 45 Gy RBE/two fractions (= 11.25 Gy RBE/fraction from 0° and 90° or 270°, respectively) delivered to the respective FTVs using each uniform field for lung and liver treatments, respectively. Carbon ion beam dose distributions were calculated as a function of respiratory cycle using optimized weight maps derived from the repeated first respiratory cycle data (planning dose) (e.g. red curve in Fig. 1d) using 4DCT data. All dose calculations were performed using the grid size of the original CT pixel size: 0.78 mm (anterior–posterior) × 0.78 mm (left–right) × 1.0 mm (superior–inferior). Respiratory cycles for planning are listed in Table 1. Dose distributions at respective phases were registered to that at the reference phase using B-spline-based DIR to create transformation maps for each voxel. Accumulated dose values for each phase were then accumulated on the basis of the respiratory-weighting function, which was extracted from the respiratory-sensing system [12]. All 4DCT data were transferred to a workstation and used to calculate 4D dose distributions using the Aqualyzer system [13].

#### Treatment dose distribution

**Fig. 2.**
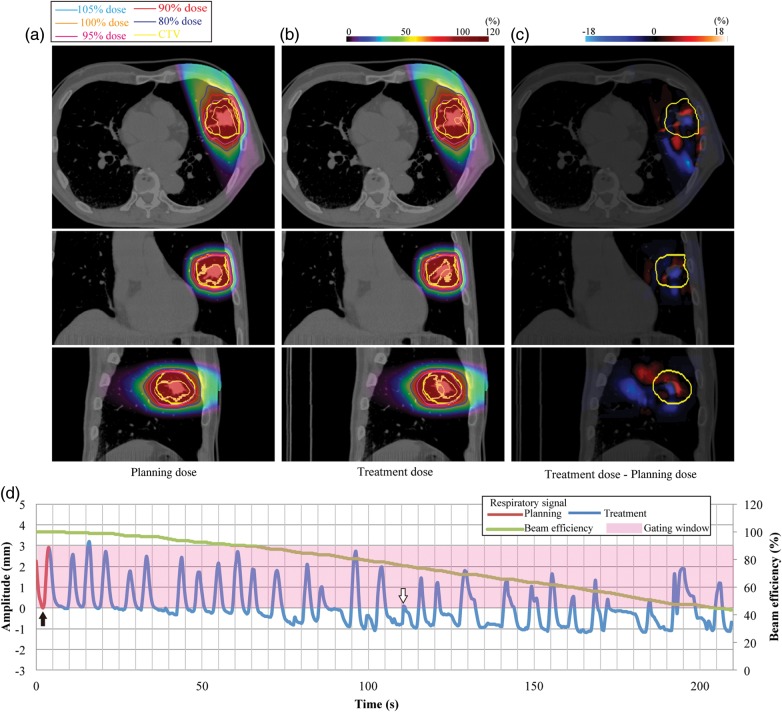
Lung dose distributions from 340° for (**a**) planning dose, (**b**) treatment dose, and (**c**) dose differences (Patient no. 1). (**d**) Respiratory wave data. Planning respiratory cycle: 4.1 s, treatment respiratory cycle: 7.4 ± 2.7 s.

**Fig. 3.**
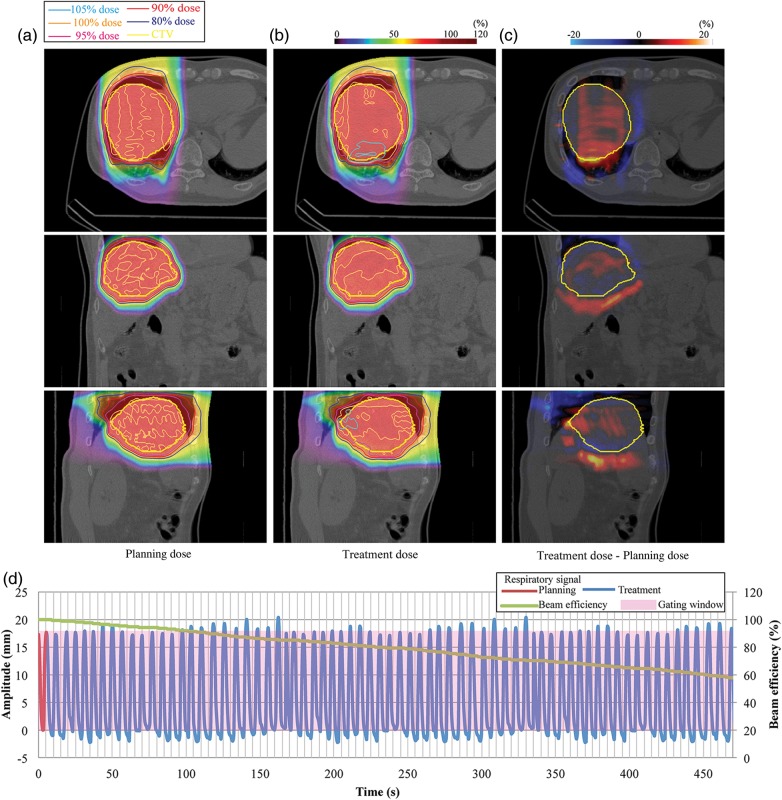
Liver dose distributions from 0° for (**a**) planning dose, (**b**) treatment dose, and (**c**) dose differences (Patient no. 17). (**d**) Respiratory wave data. Planning respiratory cycle: 5.8 s, treatment respiratory cycle: 6.8 ± 0.7 s.

**Fig. 4.**
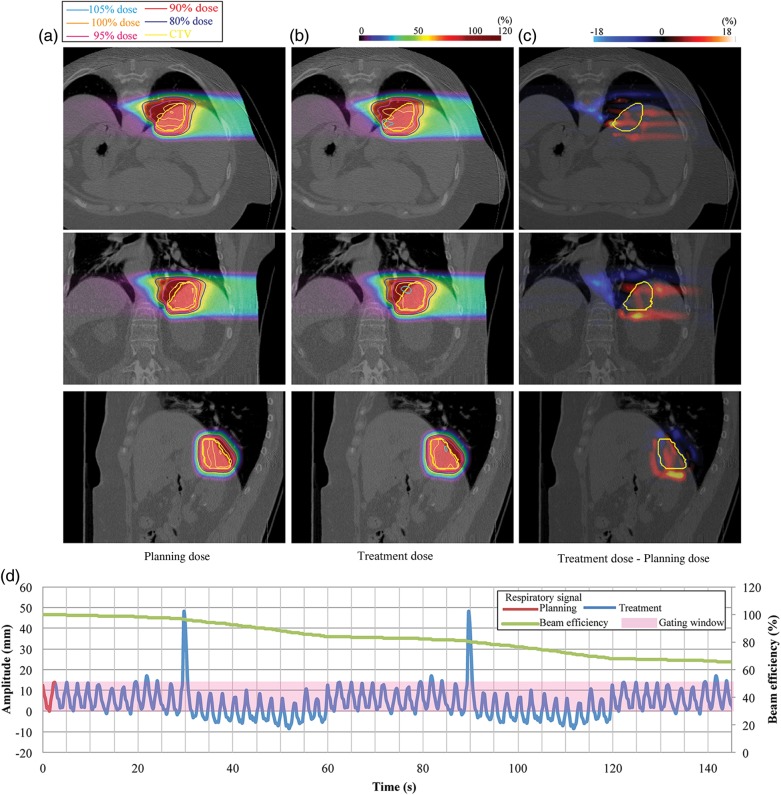
Liver dose distributions from 90° for (**a**) planning dose, (**b**) treatment dose, and (**c**) dose differences (Patient no. 18). (**d**) Respiratory wave data. Planning respiratory cycle: 2.8 s, treatment respiratory cycle: 2.6 ± 0.2 s.

Carbon ion beam dose distributions (treatment dose) were calculated as a function of respiratory cycle using the whole respiratory data, which were obtained using the same external respiratory-sensing system and same position as when 4DCT was acquired (e.g. blue curve in Fig. 1d). Respiratory cycles for treatment and respiratory pattern variations (peak inhalation and peak exhalation positions compared with those in the first respiratory cycle) are summarized in Table 1. Since all patients were actually treated with a passive scattering beam, and these respiratory data of respective patients were obtained during treatment, these data were repeated to calculate the treatment dose when scanning beam irradiation time was longer than that for passive scattering. We applied an amplitude-based gating strategy, with the gating window defined not by respiratory phase, but rather by tumor position in the 4DCT (e.g. pink region in Fig. 1d). This gating strategy is useful in avoiding unexpected breathing patterns during treatment. The gating window is not always smaller than the actual amplitude of motion (see Figs 1d, 2d, 3 and 4d). Tumor position outside the gating window (tumor displacement during 4DCT acquisition) is therefore not required for the calculation of treatment dose. The respiratory data during treatment were normalized by setting the respiratory cycle and amplitude for the first respiratory cycle to those for the 4DCT acquisition. We assumed in this study that respiratory data correlated well with tumor position. Treatment dose calculation relies on the correlation of the actual tumor motion amplitude to the best-fitting 4DCT images. Because tumor positions at mid-inhalation and mid-exhalation are not always the same, even though lung air volume is the same, the best-fitting CT image at end-exhale (black arrow in Fig. 2d) might be the 4DCT image corresponding to inhalation (white arrow in Fig. 2d) at treatment planning, and vice versa for the best-fitting CT image at end-inhale. Since tumor movement in most cases follows a 3D rather than a 2D trajectory, we also considered the respiratory phase as possible determinant. In the example shown in Fig. 1d, when the CT data selected the tumor position at 40 s (point *c*, tumor displacement of 2 mm, end-exhale), we assigned the CT data around 1 s (point *a*, tumor displacement of 2 mm, mid-exhale) rather than 3 s (point *b*, tumor displacement of 2 mm, mid-inhale) to calculate treatment dose, because points *a* and *c* were in the same exhale phase.

#### Dose assessment

We did not calculate dose summation with different beam angles. The following metrics were studied using each beam angle to assess the dose D_95_ (lowest dose encompassing 95% of the target volume), maximal (D_max_) and minimal (D_min_) dose within the CTV, as well as the dose to the cord and heart. Further, a homogeneity index (HI) was calculated as the standard deviation of the accumulated dose within the CTV. Other important parameters considered were total treatment time and beam efficiency (as defined by the ratio of beam irradiation time to total treatment time).

## RESULTS

We calculated dose distributions for tumors located in the lung and liver. Irregular motion patterns were chosen to reflect the most common respiratory features observed among our patients: in addition to a reasonably regular pattern, which can be considered a model case, we also sought respiratory patterns showing either drift motion or high amplitude spike motion.

### Tumors in the lung

Figure 1a shows the dose distribution calculated in treatment planning using the respiratory motion displayed in red in Fig. 1d for Patient no. 13. The second panel (Fig. 1b) shows the treatment dose that would be delivered under the assumption of an irregular motion pattern, namely motion drift in the superior direction (blue curve on Fig. 1d). The respiratory pattern for this can be seen in Fig. 1 for a drift case, which leads to quite poor efficiency. Planning dose distribution satisfactorily delivered over 95% of the prescribed dose to the CTV (Fig. 1a). Hot spots (>105%) within the CTV were observed during the treatment dose (Fig. 1b), and higher and lower treatment doses not seen in the planning doses were distributed in the respective superior and inferior sides of the CTV (Fig. 1c). Beam efficiency at the end of irradiation was reduced to 66% of that of the planning dose, and treatment time was 54 s longer.

We performed similar calculations for another patient (Patient no. 1) who showed a rather irregular respiratory pattern combined with drift (Fig. 2). This led to almost no irradiation in the second half of the beam delivery, and the magnitude of respiratory cycle variation was large (Patient no. 1). D_max_ and HI values (102.0% and 1.7%) in the planning dose were 1% and 0.1% smaller than those in the treatment dose, respectively. D_95_ and D_min_ values (95.0% and 89.0%) in the planning and treatment doses were the same. Since beam efficiency was reduced to 43% at the end of irradiation, treatment time was extended by 233 s from that of the planning dose (= 98.5 s).

Dose assessment metrics (D_95_, D_max_, D_min_ and HI of the CTV) are summarized in Table 2. The CTV–D_95_ value of the treatment dose (= 96.0 ± 1.0%) was ∼ 0.6% smaller than that of the planning dose (= 96.6 ± 0.9%). The CTV–D_max_ and CTV–D_min_ values of the treatment dose (= 104.5 ± 2.2% and 89.4 ± 2.6%) were slightly degraded over those of the planning dose (= 102.1 ± 1.0% and 89.8 ± 2.5%) as a result of hot spots. D_max_ values of the treatment dose for both the cord (= 0.8 ± 2.0 Gy RBE, median: 0 Gy RBE, range: 0.0–9.2 Gy RBE) and heart (= 5.2 ± 5.0 Gy RBE, median: 3.5 Gy RBE, range: 0.0–13.4 Gy RBE) were slightly increased from the respective planning dose values of 0.7 ± 1.6 Gy RBE (median: 0 Gy RBE, range: 0.0–6.7 Gy RBE) and 5.1 ± 4.9 Gy RBE (median: 3.5 Gy RBE, range: 0.0–12.2 Gy RBE). Average treatment time of the treatment dose was 43.3 s longer than that of the planning dose.

**Table 2.** Summary of dose assessment metrics averaged over all single fields

		Metrics	Mean	S.D.	Median	Range
Lung	Planning dose	CTV–D_95_ (%)	96.6	0.9	96.6	(94.2–98.0)
		CTV–D_max_ (%)	102.1	1.0	102.0	(101.0–107.0)
		CTV–D_min_ (%)	89.8	2.5	90.0	(83.0–95.0)
		CTV–HI (%)	1.2	0.3	1.2	(0.6–1.9)
		Cord–D_max_ (Gy RBE)	0.7	1.6	0.0	(0.0–6.7)
		Heart–D_max_ (Gy RBE)	5.1	4.9	3.5	(0.0–12.2)
		Time (s)	79.3	33.9	72.8	(25.3–201.6)
	Treatment dose	CTV–D_95_ (%)	96.0	1.0	96.1	(93.3–97.8)
		CTV–D_max_ (%)	104.5	2.2	104.0	(102.0–113.0)
		CTV–D_min_ (%)	89.4	2.6	90.0	(82.0–94.0)
		CTV–HI (%)	1.7	0.4	1.6	(1.0–2.9)
		Cord–D_max_ (Gy RBE)	0.8	2.0	0.0	(0.0–9.2)
		Heart–D_max_ (Gy RBE)	5.2	5.0	3.5	(0.0–13.4)
		Time (s)	122.6	70.4	105.7	(33.9–340.9)
Liver	Planning dose	CTV–D_95_ (%)	98.2	0.3	98.2	(97.2–98.7)
		CTV–D_max_ (%)	103.2	1.0	103.0	(102.0–105.0)
		CTV–D_min_ (%)	92.2	2.4	92.0	(87.0–96.0)
		CTV–HI (%)	0.7	0.2	0.7	(0.4–1.1)
		Cord–D_max_ (Gy RBE)	1.9	2.9	0.0	(0.0–9.2)
		Esophagus–D_max_ (Gy RBE)	3.7	6.1	0.0	(0.0–17.6)
		Time (s)	144.3	54.3	130.3	(57.0–272.2)
	Treatment dose	CTV–D_95_ (%)	96.9	0.9	97.0	(94.9–98.1)
		CTV–D_max_ (%)	107.8	3.5	106.5	(104.0–116.0)
		CTV–D_min_ (%)	90.6	2.3	91.0	(86.0–94.0)
		CTV–HI (%)	1.6	0.6	1.4	(0.7–3.3)
		Cord–D_max_ (Gy RBE)	1.8	2.9	0.0	(0.0–9.2)
		Esophagus–D_max_ (Gy RBE)	3.5	5.8	0.0	(0.0–16.9)
		Time (s)	210.2	109.6	179.3	(57.0–473.3)

### Tumors in the liver

The planning dose distributions from 90° delivered over 95% of the prescribed dose to the CTV without any hot spots (Fig. 3a) (Patient no.17). Figure 2d shows that respiratory motion was quite regular and remained regular for most of the time within the gating window, which was set to the minimal and maximal extension observed on treatment planning. However, hot spots in treatment dose distribution were observed around the diaphragm region, because this region experienced a large density change as a result of intrafractional replacement within the liver and lung regions (Fig. 3b). Beam efficiency was 57.8% and was affected by treatment time (planning dose, 272 s; treatment dose, 472 s).

The next liver case is shown in Fig. 4 (Patient no.18). The respiratory pattern showed a drift to the inferior side and a single sharp inhalation peak. Since less respiratory data were obtained in passive beam treatment than in scanning irradiation, the respiratory data were repeated, and the spike pattern was accordingly observed twice (Fig. 4d). A small hot spot around the distal edge of the target was seen with the treatment dose. D_95_, D_max_, D_min_ and HI for planning and treatment doses were 97.2%/95.1%, 102.0%/114.0%, 87.0%/90.0% and 1.1%/3.3%, respectively.

Liver dose assessment metrics averaged over all patients are listed in Table 2. D_95_ values of the treatment dose (= 96.9 ± 0.9%) were less than those of the planning dose (= 98.2 ± 0.3%). The CTV–D_max_ and CTV–D_min_ values of the treatment dose were degraded by 4% and 1% less than those of the planning dose, respectively, whereas the maximum D_max_ value among all patients reached 116%. The CTV–HI value of the treatment dose was increased 0.9% on average from that for planning (= 0.7 ± 0.2%). Esophagus–D_max_ (= 3.5 ± 5.8 Gy RBE, median: 0 Gy RBE, range: 0.0–16.9 Gy RBE) and cord–D_max_ (= 1.8 ± 2.9 Gy RBE, median: 0 Gy RBE, range: 0.0–9.2 Gy RBE) values of the treatment dose were higher than those of the planning dose. Average treatment time was 65.9 s longer than the planning dose.

## DISCUSSION

In this study, we showed that dose distributions for lung and liver cases with irregular respiratory patterns (treatment dose) were very similar to those of cases with regular patterns (planning dose). However, small hot spots were observed due to a residual interplay effect resulting from incomplete PCR. These findings suggest that multiple-PCR with amplitude-based gating preserves dose conformation to a moving target, even in the presence of irregular respiration.

Several publications have reported scanning particle beam distribution for moving targets and evaluated methodologies to reduce the dosimetric influence of interplay [12, 14–16]. The PCR approach provides good and robust dose distribution to a moving target under a regular breathing pattern [8, 17]. A high rescanning number with phase-control minimized the interplay effect by improving uniformity of the positional probability density; as a result, the slight differences in motion trajectory, respiratory amplitude and other factors probably led to an additional smoothing effect. Even with PCR, some interplay effect was still observed for irregular respiratory patterns. As shown by the dose assessment results, however, its influence on dose distribution was acceptable from a clinical point of view.

Most treatment centers currently use a phase-based respiratory gating strategy via the monitoring of patient thoracic/abdominal surface motion. However, information from patient surface motion cannot be used to directly monitor tumor position. If phase-based gating is applied, however, an irregular respiratory pattern may cause the tumor to move out of the treatment beam because tumor position is not always the same within the same respiratory phase. In Fig. 2d, for example, tumor positions at exhalation around 10 s and 100 s after starting treatment are not the same. Further, beam range can vary due to differences in respiratory amplitude, even in the same respiratory phase [18]. Moreover, the beam weight map was designed to give the prescribed dose to the CTV under the assumption that tumor position in treatment planning and during irradiation is the same. As a result, the prescribed dose cannot be satisfactorily delivered to the target, and excess dose to normal tissues might be increased. The basic concept of gating treatment is that the beam is on when the tumor is positioned within the gating window, as defined in the treatment planning. This condition can also be met by an amplitude-based gating strategy, which might also improve gating treatment accuracy, even though the correlation between patient surface and tumor position is low [19]. We have installed an X-ray fluoroscopic imaging system in our treatment room for clinical use with an amplitude-based gating strategy [2]. The respiratory gating system during treatment and 4DCT image acquisition should be same, however, it is impossible to acquire both CT and fluoroscopic images at the same time as a consequence of blocking X-rays from the fluoroscopic system by CT gantry. Therefore, we used the external respiratory-sensing system.

In this study, we used a gating window of 100% of the motion amplitude during 4DCT acquisition to emphasize tumor motion and did not investigate dose assessment with a narrower gating window, such as T40–T60. We expect that the application of a narrower gating window would minimize excess doses to normal tissues.

In our 24 lung and liver patients, our evaluation indicated that the prescribed dose was satisfactorily delivered to the target with > 94% of D_95_ values under irregular respiratory patterns. The beam spot map was based on information on both respiratory cycle and tumor position obtained from 4DCT. Tumor positions were within the gating window, although hot spots were observed in several cases. Variation in respiratory amplitude leads to a difference in the probability density function of the tumor position for treatment planning and delivery, and hence affects the accumulated dose distribution, as seen in Fig. 1b. If the respiratory cycle is changed from that in the treatment planning not all beam spots will be completed by the end of the gating window, which in turn means that PCR will not be fully achieved. Dose to these remaining spots will be delivered at the start of the next gating, followed by energy variation during the gating window, which will further prolong treatment and desynchronize PCR. The gating window is hence typically defined using the average respiratory curve acquisition of the planning 4DCT. The gating window during 4DCT acquisition is not exactly the same as that during irradiation as a result of interfractional changes.

One limitation of this study warrants mention. We assumed that 1D respiratory data obtained using the external respiratory monitor correlated well with tumor position. In most cases, however, intrafractional tumor movement follows a 3D trajectory, and this simplified simulation approach (characteristics or irregular motion) may have affected the results. In this regard, a method for retrospectively evaluating dose distribution during irradiation using actual patient CT data is not available: first, 4DCT imaging during whole-treatment beam irradiation increases patient radiation dose; and second, even though tumor position is acquired in 3D space during treatment beam irradiation by two X-ray fluoroscopic units, feeding this information back to the treatment planning CT image is difficult. Previous studies have typically investigated motion mitigation methods under the assumption of a regular respiratory pattern. Our present evaluation methodology is, therefore, one approach to the retrospective evaluation of dose assessment during irradiation. Third, since registration accuracy could affect the accumulated dose distribution, DIR is a key calculation technique in 4D treatment planning. DIR can be greatly affected by 4DCT artifacts (banding artifacts, missing slices etc.). The registration error could not be removed completely in the current DIR technique, especially for patient data [20], and the registration accuracy average of 1.5 mm was retained in our study. This is the caveat in the current 4D treatment planning, and oncologists and medical physicists should take it into account.

## CONCLUSION

We evaluated carbon-ion scanning beam distribution for an irregular respiratory pattern and compared results with those for a regular respiratory pattern using lung/liver 4DCT data. Using the amplitude-based gating strategy with multiple rescannings, dose distribution to the target was preserved, even though respiratory pattern was irregular. Nevertheless, the amplitude-gating strategy alone would be not sufficient for other scanned beam delivery systems (e.g. specification of the beam delivery system and/or no PCR). Our results provide useful information that closely reflects clinical situations, particularly in scanning treatment, which is sensitive to organ motion. We are ready to commence clinical trials of carbon-ion scanning beam therapy for the thoracic and abdominal regions and expect the results to be beneficial to our patients, our center and the particle therapy community.
